# Cracking the fertility code: a comprehensive review of biomarkers and functional measures

**DOI:** 10.1098/rsob.260199

**Published:** 2026-10-21

**Authors:** Morgan F. Woodman-Sousa, Kathryn Grive

**Affiliations:** ^1^ Molecular Biology, Cell Biology, and Biochemistry Graduate Program, Brown University, Providence, RI, USA; ^2^ Obstetrics and Gynecology, Women & Infants Hospital of Rhode Island, Providence, RI, USA; ^3^ Obstetrics and Gynecology, Brown University Warren Alpert Medical School, Providence, RI, USA

**Keywords:** fertility, biomarker, endocrinology, infertility, reproductive medicine

## Abstract

Human fertility is regulated by a complex, integrated network of biological systems that collectively determine reproductive competence in males and females. Conventional fertility assessment relies on established clinical biomarkers, including reproductive hormone profiles, gamete-based functional assessments and imaging indicators of gonadal structure and function. While clinically informative, these measures capture only partial views of molecular and cellular processes underlying gametogenesis and reproductive capacity. Recent advances in reproductive medicine have expanded the biomarker landscape through genetic and epigenetic profiling, enabling identification of pathogenic variants, chromosomal abnormalities and regulatory modifications associated with infertility syndromes. In parallel, multi-omics approaches are increasingly being used to characterize molecular networks governing gamete development, meiotic integrity, DNA repair and cellular differentiation. This review presents a comprehensive synthesis of fertility biomarkers, spanning endocrine, genetic, epigenetic, structural, metabolic and inflammatory systems. Current evidence is examined on endocrine and reproductive hormone biomarkers, followed by an investigation of genetic and epigenetic markers of reproductive dysfunction, as well as structural and imaging-based biomarkers and inflammatory and metabolic indicators. Finally, emerging biomarker platforms are highlighted as key advances towards precision reproductive medicine. Collectively, these developments support a shift towards a more comprehensive, biomarker-driven framework for the evaluation and management of infertility.

## 1. Introduction

Infertility affects roughly one in six adults worldwide (≈17.5%), making it one of the most common yet under-recognized global health challenges today [1]. Clinically defined as the failure to achieve a clinical pregnancy after 12 months or more of regular, unprotected intercourse, it is recognized by the World Health Organization as a disease of both the male and female reproductive systems [1]. Beyond its biological definition, infertility is often associated with significant psychological distress, pervasive social stigma, financial strain and reduced quality of life and mental well-being. Yet, despite its prevalence, approaches to the prevention, diagnosis and treatment of infertility remain limited, underfunded and not universally accessible. Thus, the identification of reliable and mechanistically informative biomarkers is crucial for advancing our understanding of infertility, improving diagnostic measures and guiding targeted treatment strategies in both males and females. This comprehensive review evaluates infertility-associated biomarkers and clinical assessments in both sexes, highlighting key gaps and emerging strategies for addressing infertility in reproductive medicine. To contextualize the concepts explored in this review, the basic structure and function of the ovaries and testes are first outlined.

The ovary is a central organ in female reproduction, responsible for oocyte production and the regulation of reproductive hormones (figure 1A). It is organized into a cortex and medulla, with the cortex containing follicles at various stages of development that support cyclic folliculogenesis and endocrine function [2]. The functional unit of the ovary is the ovarian follicle, a highly coordinated structure composed of a centrally located oocyte surrounded by specialized somatic cells [3–6]. The oocyte is enclosed by granulosa cells, which provide metabolic support, regulate meiotic arrest and produce oestrogen, a hormone essential for follicular maturation and the hormonal regulation of ovulation [7]. Through extensive bidirectional communication with the oocyte, granulosa cells also mediate paracrine signalling essential for follicular growth and survival [7]. As follicles develop, they acquire surrounding theca cells and become increasingly regulated by endocrine signalling through the hypothalamic–pituitary–gonadal (HPG) axis [8]. Follicles mature through primordial, primary, secondary, antral and preovulatory stages, with increasing structural complexity and eventual ovulation followed by corpus luteum formation (figure 1B) [6]. Concurrently, oogenesis begins during fetal development, when oocytes enter meiosis and become arrested in prophase I, where they remain throughout follicular quiescence as part of the ovarian reserve [6]. Although follicles are continuously recruited for growth after puberty, the enclosed oocytes remain meiotically arrested until the preovulatory luteinizing hormone (LH) surge induces meiotic resumption [6]. The oocyte then completes meiosis I, extrudes the first polar body and arrests again at metaphase II before ovulation, completing meiosis II only upon fertilization [6].

**Figure 1.**
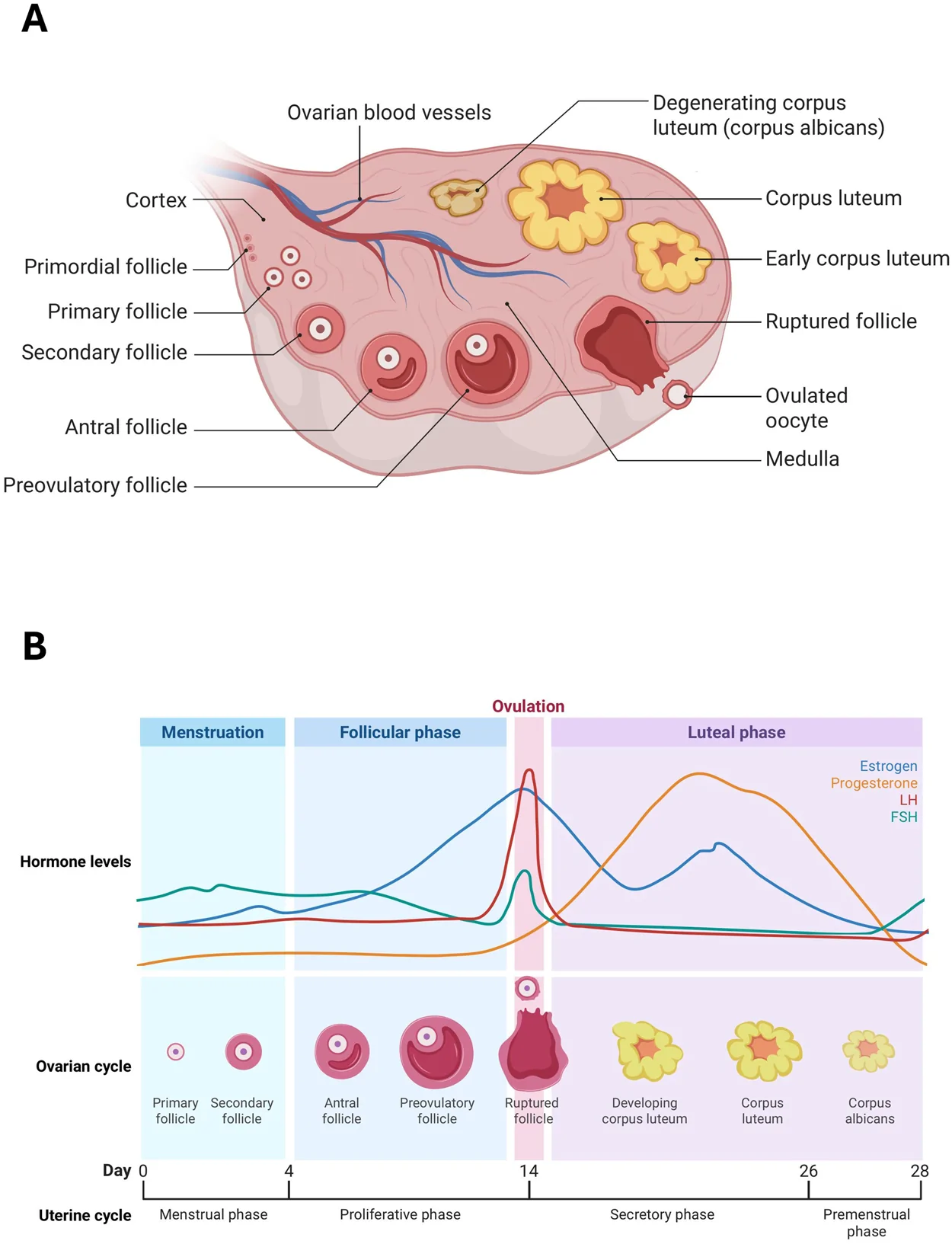
Representative images of (A) ovarian structure and (B) folliculogenesis, including the ovarian and uterine cycles and associated fluctuations in reproductive hormone levels (created in BioRender. Woodman (2026) https://BioRender.com/tf6y66r).

Within this structural framework, female reproductive capacity is determined by a finite pool of oocytes established during fetal development, known as ovarian reserve. Female infants are born with approximately 500 000 to one million oocytes, and this reserve declines progressively over time owing to continuous follicular atresia and cyclic ovulation, ultimately culminating in menopause [9]. Ovarian reserve refers to the quantity of oocytes, whereas oocyte quality refers to the developmental competence of an oocyte to support fertilization and result in a live birth. While ovarian reserve declines relatively consistently with advancing age, age-associated declines in oocyte quality are more heterogeneous. Nevertheless, considerable inter-individual variability exists among women of the same chronological age in both the magnitude and timing of declines in oocyte quantity and quality [9,10].

In males, the testes are the primary reproductive organs, located inside the scrotum (figure 2). Within the testes, spermatogenesis occurs in highly structured seminiferous tubules and, unlike oogenesis with its finite ovarian reserve, continues throughout the adult lifespan following puberty, providing an ongoing source of sperm production [11]. Each seminiferous tubule contains a specialized germinal epithelium composed of developing germ cells at successive stages of differentiation, supported by Sertoli cells, which provide structural organization, metabolic support and paracrine regulation essential for germ cell survival and maturation [12]. Sertoli cells also form the blood–testis barrier, a specialized tight junction divider that creates a protected microenvironment for meiotic and post-meiotic germ cells. Within the interstitial space between seminiferous tubules, Leydig cells produce testosterone, which is essential for the initiation and maintenance of spermatogenesis as well as the development of male reproductive maturation [11].

**Figure 2.**
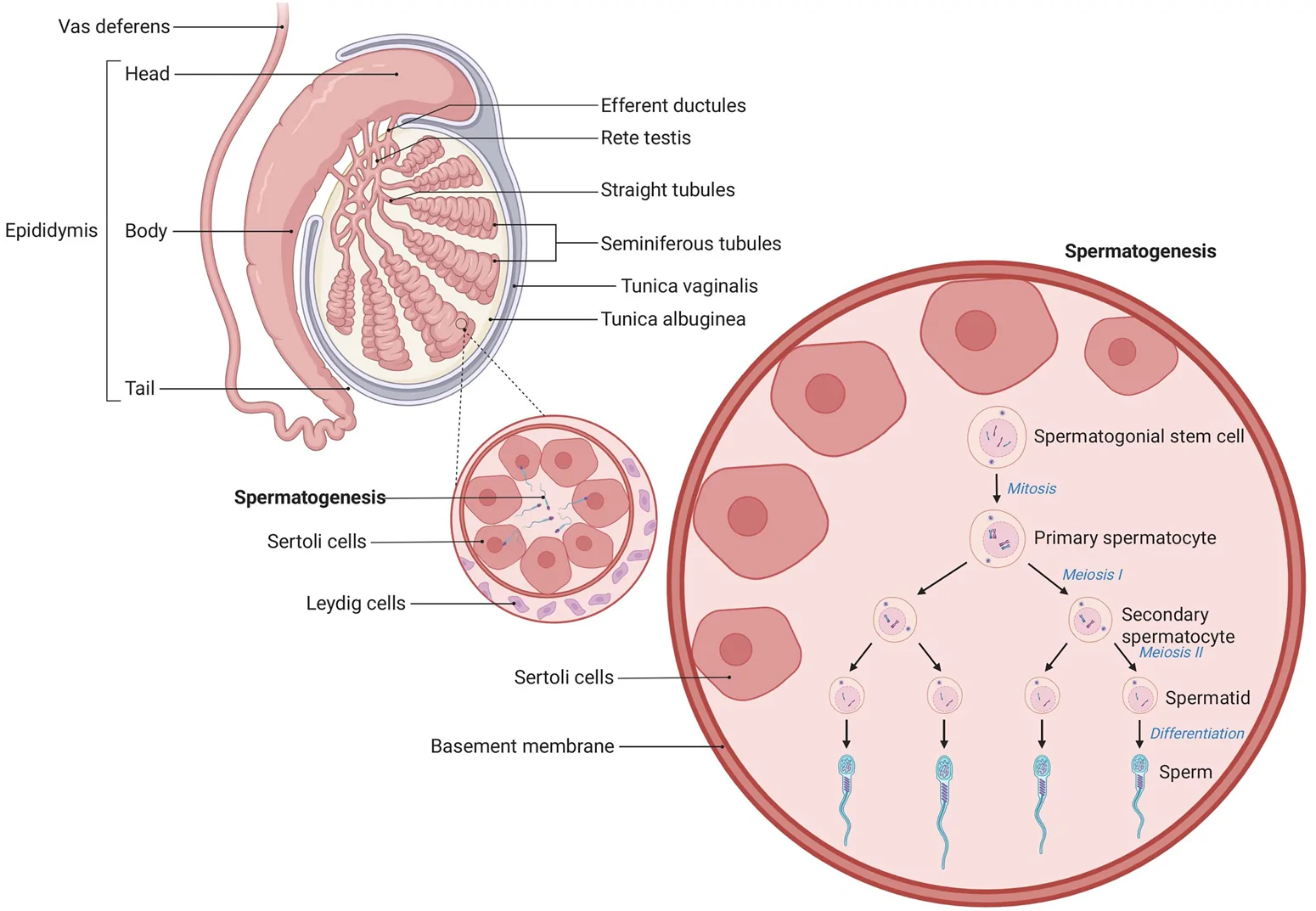
Representative image of testicular anatomy, highlighting the site of spermatogenesis (Created in BioRender. Woodman (2026) https://BioRender.com/tf6y66r).

Spermatogenesis is the highly orchestrated process through which spermatogonia differentiate into mature spermatozoa. The process is tightly regulated by endocrine signalling via the HPG axis, which modulates testosterone and gonadotropin levels to maintain spermatogenic homeostasis [11]. Beginning with spermatogonial stem cells situated along the basement membrane of the seminiferous epithelium, germ cells undergo successive mitotic and meiotic divisions through the primary and secondary spermatocyte stages, ultimately producing haploid spermatids [12]. Spermatids will then undergo spermiogenesis, a maturation process involving nuclear condensation, acrosome formation and flagellar development, resulting in fully differentiated spermatozoa that are released into the tubular lumen [11,12].

Following spermiation, spermatozoa are transported through the rete testis and efferent ductules to the epididymis, where they acquire motility and fertilization competence. Mature sperm are subsequently transported via the vas deferens, a muscular tube that mediates rapid sperm transport during ejaculation and connects the epididymis to the ejaculatory ducts [13].

Despite clear anatomical and functional differences, the male and female reproductive systems are unified by a shared organizational framework in which germ cell development is supported by specialized somatic microenvironments and coordinated endocrine regulation. Disruption of these tightly regulated networks can impair reproductive function.

Currently, infertility evaluation relies on a combination of clinical history, hormonal assessment, imaging and laboratory-based testing to identify abnormalities affecting reproductive function. However, diagnostic criteria differ across infertility-associated disorders, reflecting the diverse biological mechanisms underlying impaired fertility. Although these approaches remain essential for clinical diagnosis and management, they often provide limited insight into the molecular and cellular processes contributing to reproductive dysfunction, highlighting the need for improved biomarkers and more comprehensive assessment strategies.

Together, these biological foundations and clinical considerations provide the framework for the subsequent discussion of infertility-associated biomarkers and clinical reproductive health assessments in both sexes.

## 2. Endocrine regulation and indicators of reproductive function

In both sexes, reproduction and fertility are tightly regulated by hormones of the HPG axis (figure 3A,B). Serum-based hormonal biomarkers offer quantifiable measures of this HPG activity and serve as clinical tools for evaluating fertility and fecundity outcomes (tables 1 and 2; a summary of all current diagnostic criteria for major infertility-associated conditions is provided in table 3). In females, these endocrine markers are valuable for estimating ovarian reserve and anticipating oocyte yield with controlled ovarian stimulation, yet they remain poor predictors of reproductive potential when considered independently of age. Likewise, in males, hormonal markers provide insight into testicular function and spermatogenesis, but they are insufficient as isolated predictors of fertility outcomes. Accordingly, hormonal biomarkers should be interpreted alongside age, clinical history and functional reproductive assessments.

**Figure 3.**
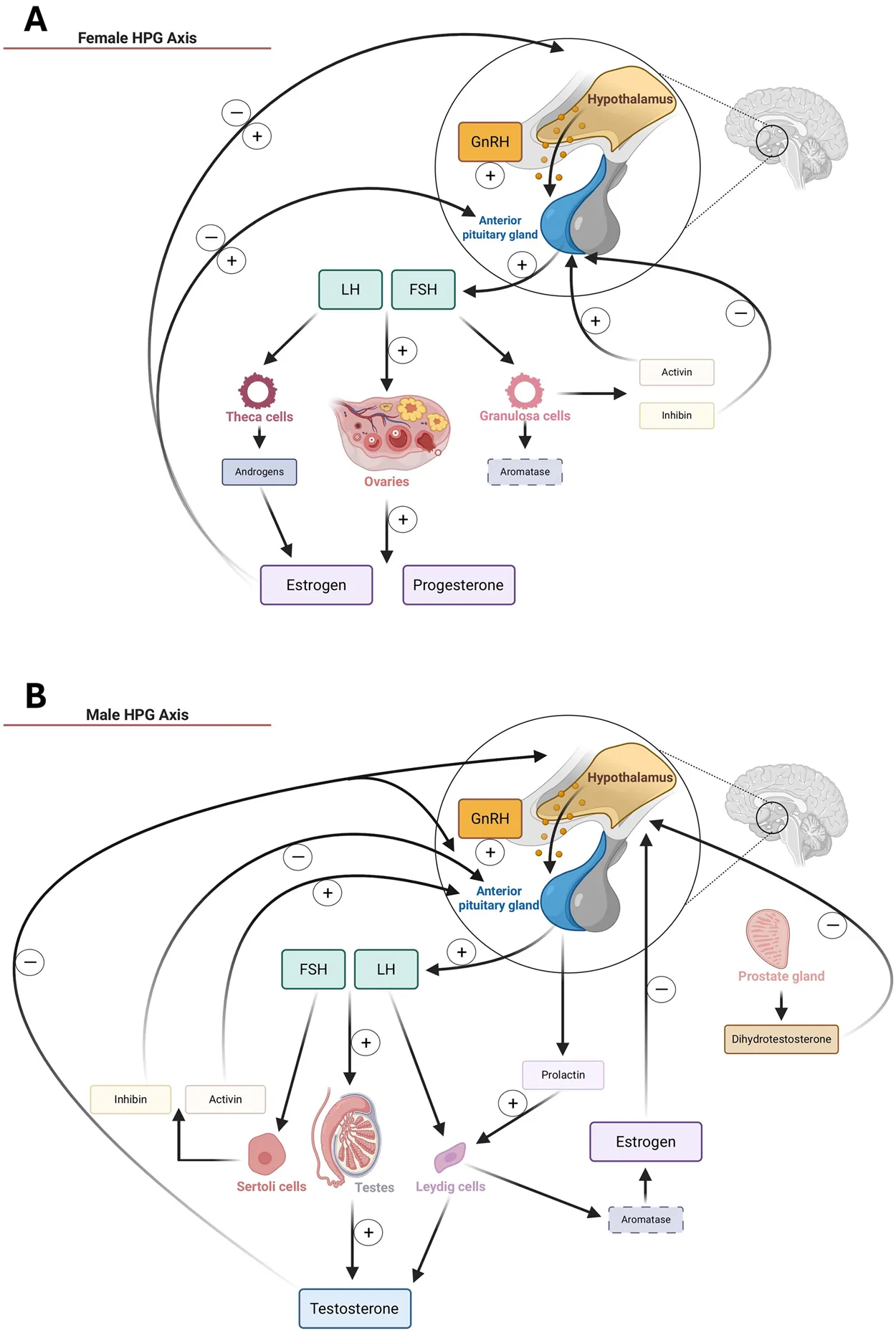
Schematic of sex-specific hormone production within the HPG axis in females (A) and males (B) (Created in BioRender. Woodman (2026) https://BioRender.com/tf6y66r).

**Table 1.** Overview of reproductive endocrine markers in females, highlighting source, physiologic significance and clinical utility in fertility evaluation.

hormone	primary source	what it reflects	clinical utility for fertility	key notes / limitations
**anti-Müllerian hormone**	granulosa cells (pre-antral and small antral follicles)	ovarian reserve (quantity)	best single marker of ovarian reserve; predicts ovarian response to stimulation	does not directly predict natural conception; assay variability
**FSH (cycle day 2–3)**	pituitary	follicular recruitment (indirect)	elevated levels indicate diminished ovarian reserve (DOR)	cycle-dependent; less sensitive than AMH
**oestradiol (cycle day 2–3)**	granulosa cells	early follicular activity	helps interpret FSH; elevated early E2 may mask high FSH	poor standalone marker
**inhibin B**	granulosa cells	follicular activity and FSH feedback	reflects declining ovarian reserve	high variability; limited routine use
**LH**	pituitary	ovulation and theca cell function	useful for predicted ART outcomes	not a reserve marker
**progesterone (mid-luteal)**	corpus luteum	ovulation confirmation	confirms ovulation and luteal function	not predictive of reserve
**prolactin**	pituitary	hypothalamic–pituitary regulation	identifies secondary causes of infertility	only relevant when abnormal
**androgens**	ovaries/adrenals	follicular environment	useful in PMOS/PCOS evaluation	indirect fertility relevance

**Table 2.** Overview of reproductive endocrine markers in males, highlighting source, physiologic significance and clinical utility in fertility evaluation.

hormone	primary source	what it reflects	clinical utility for fertility	key notes / limitations
**FSH**	pituitary	Sertoli cell function (indirect)	strong predictor of impaired spermatogenesis; elevated in primary testicular failure and non-obstructive azoospermia	not perfectly correlated with focal spermatogenesis
**inhibin B**	Sertoli cells	direct marker of spermatogenic activity	closely correlates with sperm count and testicular histology	assay variability; less standardized globally
**testosterone**	Leydig cells	androgen support of spermatogenesis	essential for normal sperm production	serum levels may not reflect intratesticular testosterone
**LH**	pituitary	Leydig cell stimulation	helps distinguish primary versus secondary hypogonadism	indirect effect on spermatogenesis
**AMH**	Sertoli cells	Sertoli cell maturity/function	adjunct marker; higher relevance in pediatrics	limited value in adult fertility assessment
**oestradiol**	peripheral aromatization	androgen–oestrogen balance	useful in selected cases (obesity, hormonal imbalance)	not a primary fertility marker
**prolactin**	pituitary	hypothalamic–pituitary regulation	screens for secondary causes of infertility	only relevant when elevated

**Table 3.** Current diagnostic criteria for major infertility-associated disorders. Abbreviations: AMH, anti-Müllerian hormone; AFC, antral follicle count; BBT, basal body temperature; DHEAS, dehydroepiandrosterone sulfate; FSH, follicle stimulating hormone; HSG, hysterosalpingography; LH, luteinizing hormone.

disorder	diagnostic criteria*	characteristic biomarker profile	associated clinical presentation
**primary ovarian insufficiency (POI)**	• amenorrhea/oligomenorrhea ≥4 months before age 40 • elevated FSH on two samples ≥4 weeks apart (typically >25–40 mIU ml^–1^) • low oestradiol (<50 pg ml^–1^) • exclude pregnancy, prolactin and thyroid disorders [14,15]	FSH ↑, oestradiol ↓, AMH ↓, AFC ↓	vasomotor symptoms, infertility, irregular menses, oligomenorrhea, amenorrhea for 4–6 months, low oestradiol levels and elevated FSH
**DOR**	• reduced ovarian reserve relative to age • identified through ovarian reserve testing [16]	AMH ↓, AFC ↓, FSH ↑	infertility, abnormal-but-not-postmenopausal ovarian reserve test results, reduced response to ovarian stimulation; menstrual cycles may remain regular
**polyendocrine metabolic ovarian syndrome (PMOS)/ polycystic ovary syndrome (PCOS)**	• at least two of the following three criteria (after exclusion of other etiologies): ovulatory dysfunction (oligo-/anovulation); clinical and/or biochemical hyperandrogenism; polycystic ovarian morphology on ultrasound or elevated AMH in adults [17]	testosterone ↑, DHEAS ↑, AMH ↑, LH:FSH ratio ↑	oligo-/anovulation with irregular menstruation, clinical hyperandrogenism (hirsutism and acne) and obesity/metabolic dysfunction. May result in infertility and miscarriage, and is associated with adverse pregnancy outcomes and increased cardiometabolic, psychological and endometrial malignancy risk
**primary hypogonadism (hypergonadotropic hypogonadism)**	• symptoms/signs of testosterone deficiency • low morning serum total testosterone after an overnight fast on ≥2 occasions • elevated LH and/or FSH indicating testicular dysfunction [18]	LH ↑, FSH ↑, oestradiol/testosterone ↓	low libido, erectile dysfunction, fatigue, infertility, mood changes and muscle and bone loss
**secondary hypogonadism (hypogonadotropic hypogonadism)**	• symptoms/signs of testosterone deficiency • low morning serum total testosterone after an overnight fast on ≥2 occasions • low or non-elevated LH and FSH indicating hypothalamic/pituitary dysfunction [18]	LH ↓/normal, FSH ↓/normal, oestradiol/testosterone ↓	low libido, erectile dysfunction, fatigue, infertility, mood changes and muscle and bone loss
**hyperprolactinemia**	• elevated serum prolactin level • exclude pregnancy medications, hypothyroidism and stress • serum prolactin >250 ng ml^–1^ suggests prolactinoma; >500 ng ml^–1^ suggests macroprolactinoma [19,20]	prolactin ↑, LH ↓, FSH ↓, oestradiol/testosterone ↓	amenorrhea, galactorrhea, anovulation, infertility, erectile dysfunction, decreased facial/body hair, gynecomastia and testicular shrinkage
**luteal phase defect (LPD)**	• short luteal phase (≤10 days) • may be assessed by BBT or urinary LH • serum progesterone measurement • endometrial biopsy [21,22]	mid-luteal progesterone ↓	short luteal phase, implantation failure, recurrent pregnancy loss
**functional hypothalamic amenorrhea (FHA)**	• low oestrogen (often <50 pg ml^–1^) • low LH and FSH (often <10 mIU ml^–1^ • normal-to-low LH:FSH ratio • normal testosterone [23–25]	LH ↓, FSH ↓/normal, oestradiol ↓, ↓/normal LH:FSH ratio	amenorrhea, cold hands and fee, hair loss/hair thinning, frequent urination, low libido, low BMI, low bone density, excessive exercise, psychological stress, infertility, disordered eating
**endometriosis**	• clinical diagnosis based on characteristic symptoms, medical history and physical examination • laparoscopic confirmation may be used when needed [26]	no specific diagnostic hormonal pattern; ovarian reserve markers may be reduced in some patients	chronic pelvic pain, dysmenorrhea, dyspareunia, painful urination or bowel movements, gastrointestinal symptoms, such as bloating or constipation, fatigue, difficulty getting pregnant
**tubal factor infertility**	• tubal occlusion, hydrosalpinx or impaired tubal patency demonstrated by HSG and/or laparoscopy [27]	normal hormonal values	infertility despite regular ovulation; may be associated with pelvic/abdominal pain, dysmenorrhea or history of pelvic infection
**Sertoli cell-only syndrome (SCOS)**	• testicular histology showing seminiferous tubules lined exclusively by Sertoli cells with complete absence of germ cells [28–31]	FSH ↑, inhibin B ↓, azoospermia, LH normal/↑, testosterone normal/↓	testicular hypotrophy, gynecomastia and eunoicoid appearance, severe male-factor infertility
**nonobstructive azoospermia (NOA)**	• absence of sperm in the ejaculate; confirmed by centrifuged semen sample • owing to impaired spermatogenesis [32–35]	FSH ↑, inhibin B ↓, LH normal/↑, testosterone normal/↓, AMH ↓, ↑ prolactin	no noticeable symptoms, severe male-factor infertility
**obstructive azoospermia (OA)**	• azoospermia owing to mechanical obstruction of the male reproductive tract • preserved spermatogenesis • normal gonadotropin and testosterone [36–38]	normal hormonal values	no noticeable symptoms, severe male-factor infertility
**varicocele**	• abnormal dilatation of the pampiniform plexus in the testis • associated abnormal semen parameters [39,40]	Testosterone ↓/normal, Inhibin B ↓, abnormal semen analysis (↓ count, motility, morphology)	scrotal discomfort, asymptomatic, infertility
**congenital bilateral absence of the vas deferens (CBAVD)**	• congenital absence of bilateral vas deferens • may be associated with cystic fibrosis and other urogenital anomalies [41,42]	FSH ↓/normal, level of each seminal plasma biochemical marker ↓	male infertility, low ejaculate volume, absent vas deferens on exam

^*^Diagnostic criteria summarized from current literature, clinical practice guidelines and consensus statements; specific laboratory thresholds may vary among professional societies and institutions.

### 2.1. Anti-Müllerian hormone

#### 2.1.1. Female

AMH is currently the most widely used and among the most reliable hormonal biomarkers of ovarian reserve. It serves as an informative tool in diverse clinical contexts, including infertility treatment, particularly *in vitro* fertilization (IVF), prediction of reproductive lifespan, evaluation of ovarian dysfunction, such as PMOS, previously named PCOS, and assessment following gonadotoxic cancer therapies or ovarian surgery [43–45].

AMH is a glycoprotein that belongs to the transforming growth factor-β family. It is produced by the granulosa cells of preantral and small antral follicles and thus serves as an indirect measure of ovarian reserve as it reflects the population of actively growing antral follicles, which correlates but is not equivalent to the primordial follicle pool [46,47]. As a result, perturbations that selectively affect the growing follicle population may alter AMH levels without corresponding changes in the underlying ovarian reserve [47,48]. Consistent with this, AMH levels have been shown to be transiently influenced by iatrogenic factors, including short-term declines following ovarian cystectomy and decreases observed after uterine artery embolization or hysterectomy [47,49–51]. Serum AMH levels are independent of gonadotropin regulation and remain relatively consistent within and between menstrual cycles in both young, normally ovulating females and females with infertility, making it a reliable candidate biomarker of ovarian reserve [52–56]. AMH is one of the earliest markers for detecting DOR and poor ovarian response (POR) to ovarian stimulation; however, it naturally declines with age and correlates with time to menopause. Levels may also be reduced in those using hormonal contraceptives [56–61]. AMH is currently considered a first-line test for evaluating ovarian reserve, with 60% of surveyed IVF clinics reporting its routine use and 89% considering it relevant to clinical practice, supporting its value as a key hormonal biomarker in infertility assessment [43].

#### 2.1.2. Male

In males, AMH is secreted by Sertoli cells in the testes and is essential for normal male sexual differentiation through regression of the Müllerian ducts during fetal development. In prepubertal males, serum AMH is widely used by paediatric endocrinologists as a reliable marker of immature Sertoli cell number and function [62,63]. In adult males, however, its clinical utility as a fertility biomarker is limited, as circulating AMH primarily reflects Sertoli cell differentiation status and androgen-mediated regulation rather than active spermatogenesis [64].

In certain infertility contexts, reduced androgenic inhibition permits follicle-stimulating hormone (FSH) to upregulate testicular AMH secretion in adults, reflecting impaired Sertoli cell maturation [63]. Because AMH indirectly reflects Sertoli cell integrity and function, it has been investigated as a biomarker for predicting sperm retrieval success in males with idiopathic non-obstructive azoospermia, where it may serve as a surrogate indicator of spermatogenic capacity and blood–testis barrier integrity [62].

### 2.3. Follicle -stimulating hormone and oestradiol

#### 2.3.1. Female

FSH and oestradiol, a form of oestrogen, are two hormones routinely measured during fertility testing to assess ovarian reserve and predict response to controlled ovarian hyperstimulation [57,65]. FSH is produced by the anterior pituitary in response to hypothalamic gonadotropin-releasing hormone (GnRH) while oestradiol is produced by the granulosa cells of growing follicles [66,67]. Existing together in a tightly regulated feedback loop, these hormones measured in tandem have been used for more than 30 years as an indirect assessment of ovarian reserve [65,68,69].

At the beginning of the menstrual cycle, FSH stimulates granulosa cells to synthesize aromatase, which converts androgens into oestradiol [70]. Rising oestradiol levels exert negative feedback to the hypothalamus and pituitary, suppressing FSH secretion and ensuring that only the most responsive follicles continue to develop [71]. Basal FSH levels are measured early in the cycle (typically cycle days 2–4) when oestradiol is expected to be low [72].

As fertility potential declines, fewer follicles produce sufficient oestrogen and inhibin, prompting a compensatory rise in FSH, which is interpreted as DOR and a POR to stimulation [68]. Conversely, a normal FSH level may be falsely reassuring if oestradiol is elevated and suppressing it; therefore, oestradiol should be measured concurrently for an accurate interpretation of FSH [65,68].

Importantly, measurement of FSH and oestradiol has several limitations, including significant inter- and intracycle variability that reduces reliability and a dependence on an intact HPG axis [68,73,74]. In addition, these markers lack sensitivity for early ovarian dysfunction, as only elevated values are clinically meaningful and FSH typically does not increase until ovarian reserve has already undergone substantial decline [57,75].

#### 2.3.2. Male

Produced in the pituitary gland, FSH serves as a key blood-based biomarker for male fertility as it provides an indirect reflection of spermatogenic function and health [76,77]. FSH promotes the growth and differentiation of Sertoli cells, which are essential for facilitating spermatogenesis, the process through which sperm cells develop and mature in the testes [78]. FSH also regulates the expression of genes that govern cell junction organization, thereby influencing the transport of nutrients and regulatory substances from Sertoli cells to developing germ cells [79].

Elevated FSH levels are often indicative of primary testicular failure or severe dysfunction, owing to impaired negative feedback, and are associated with spermatogenic defects ranging from azoospermia to reduced sperm quantity and quality [80].

Elevated FSH levels may result from testicular damage (e.g. radiation exposure, trauma or chronic alcohol use), underlying medical or genetic conditions, advancing age, hormonal dysregulation, certain medications (such as opioids or anabolic steroids) or systemic diseases including HIV/AIDS and type 2 diabetes [80–84]. In rare cases, elevated FSH may be associated with pituitary tumours [85].

Low FSH levels are less common and typically suggest suboptimal pituitary function. Potential causes include excess androgen exposure, pituitary cell damage, genetic abnormalities, hyperprolactinemia and other endocrine disorders [81]. Environmental factors and exposure to specific substances may also contribute [81].

In males, oestradiol is the predominant form of oestrogen and is essential for modulating libido, erectile function and spermatogenesis [86]. It is produced primarily through the aromatization of testosterone [87,88]. Orstradiol is produced locally in multiple male reproductive cell types, including germ cells, spermatozoa, Sertoli cells, Leydig cells and the epithelium of the efferent ductules and proximal epididymis, where it exerts cell-specific functions [87,89–93]. Its actions include regulating spermatogenesis, modulating germ cell proliferation, differentiation, survival and apoptosis, influencing Sertoli and Leydig cell function and supporting fluid reabsorption in the efferent ductules to affect sperm concentration, motility and morphology [87,94–109].

Oestradiol alone is not a consistent predictor of male fertility outcomes. Its clinical value is strongest when interpreted alongside testosterone levels or, more commonly, as part of the testosterone-to-oestradiol ratio, which better reflects endocrine balance in the testes [58,110,111]. Some studies indicate that elevated levels of oestradiol or a reduced testosterone-to-oestradiol ratio is associated with impaired spermatogenesis and male infertility; however, these data are limited [86,87,112]. Mechanistically, high levels of oestradiol can suppress the HPG axis, leading to reduced LH and FSH secretion and lower intratesticular testosterone, which ultimately disrupts sperm production [113]. Hyperoestrogenism may result from medications that disrupt the HPG axis as well as from diseases of the adrenal cortex or testis [87].

### 2.4. Inhibin B

#### 2.4.1. Female

Inhibin B is produced by granulosa cells of developing follicles, and is a heterodimeric glycoprotein within the superfamily of transforming growth factor-β [114]. Inhibin B plays a key role in suppressing FSH through negative feedback on the pituitary gland [114]. In reproductive-age females, high levels of serum inhibin B maintain lower concentrations of circulating FSH [114]. However, as ovarian reserve declines with age, serum inhibin B levels decrease, weakening inhibition of FSH and contributing to the progressive rise in serum FSH observed with reproductive ageing [114–117].

For healthy reproductive age females, studies have found that serum inhibin B levels were significantly positively correlated with AMH and antral follicle count (AFC), and negatively correlated with age, FSH, LH and FSH/LH [114,118–121]. Moreover, inhibin B had an advantage over FSH in predicting AFC <5–7 mm, which primarily reflects the pool of small antral follicles (typically 2–5 mm in humans), suggesting the potential utility of inhibin B in predicting ovarian response [114,122]. These findings suggest a potential role for inhibin B in the clinical assessment of ovarian reserve and female reproductive capacity.

However, inhibin B is not currently considered a reliable marker of ovarian reserve in clinical practice for several reasons. First, inhibin B concentrations vary substantially across both the menstrual and assisted reproductive technology (ART) cycles [114]. During the early follicular phase, concentrations rise and peak shortly after the early follicular FSH rise before declining throughout the remainder of the follicular phase [114,123]. Following the midcycle LH surge, inhibin B exhibits a transient secondary increase and then remains low throughout the luteal phase [114,123,124]. Additionally, there is no standardized method to measure the absolute value of inhibin B, and thus values differ greatly across studies with differences in patient populations and assay methods [114].

#### 2.4.2. Male

In males, inhibin B is produced exclusively by Sertoli cells in the testis, and it directly reflects their function [125,126]. It provides negative feedback on FSH secretion and, in adulthood, inhibin B production is influenced by both FSH and the stage of spermatogenesis, although the exact role that germ cells play in its regulation is not fully understood [127,128]. Its regulation also varies across the lifespan; serum inhibin B shows a transient peak shortly after birth, which only partially parallels FSH levels and likely reflects heightened Sertoli cell proliferation during this period [128]. Levels then decline and remain low throughout childhood until puberty, when they rise again initially in response to FSH stimulation and subsequently through combined regulation by FSH and active spermatogenesis [128].

In adult males, circulating inhibin B exhibits diurnal variation that closely parallels testosterone and correlates positively with testicular volume and sperm concentration [126,128–130]. In infertile males, reduced inhibin B is typically accompanied by elevated FSH, and levels generally reflect the severity of impaired spermatogenesis, with the lowest concentrations observed in more severe germ cell arrest [126,128]. However, exceptions exist, including cases of Sertoli-cell-only syndrome with normal inhibin B levels [128]. Upon administration of exogenous FSH in healthy males, inhibin B secretion increases, with a more marked response observed in men with secondary hypogonadism [128,131]. However, FSH therapy in infertile men does not consistently produce a significant rise in inhibin B [128].

Various studies report that Inhibin B levels could be a potential marker for spermatogenesis and testicular and seminiferous tubule function, and serve as a useful marker of male factor infertility [126,129,131]. In males with normal sperm concentrations, inhibin B levels are significantly higher, whereas they are undetectable or reduced in sub-fertile males, with the exception of those with obstructive azoospermia or spermatogenic arrest at various stages [126,127,129,132].

Inhibin B has become an important non-invasive indicator of testicular function in clinical settings, with applications including infertility diagnostics, evaluation of pre-pubertal populations, surveillance of gonadotoxic exposures and prediction of assisted reproduction outcomes [130,133]. When used alongside semen analysis and FSH assessment, evaluating inhibin B levels can strengthen diagnostic accuracy for conditions such as age-associated declines in male fertility, varicocele-related dysfunction and non-obstructive azoospermia [130,133].

While an overall relationship exists between FSH, testicular function, particularly spermatogenesis, and inhibin B levels in blood, their complex dynamics are not fully understood and inhibin B has yet to be established as a standalone marker of male factor infertility.

### 2.5. Luteinizing hormone

#### 2.5.1. Female

LH is a glycoprotein hormone that is secreted by the pituitary gland, alongside FSH [134]. LH release is stimulated by GnRH and inhibited by oestrogen in females [135]. LH regulates the timing and progression of the female menstrual cycle, triggers ovulation, supports preparation of the uterus for implantation and stimulates the production of steroid hormones, while also contributing to germ cell maturation [136]. In the ovary, LH stimulates theca cells to produce testosterone, which neighbouring granulosa cells subsequently convert into oestrogen [136]. A pronounced surge in LH secretion, known as the preovulatory LH surge, triggers the release of a mature oocyte during ovulation, and following ovulation, the remaining follicular cells proliferate to form the corpus luteum, which secretes progesterone and oestradiol [136]. Some studies indicate that LH plays a critical role in follicle and oocyte development [137,138].

Although it is not a direct biomarker of fertility, LH levels in the body can allude to ovulatory timing and underlying dysregulation of the HPG axis, providing indirect insight into reproductive function and potential fertility outcomes. Females with low LH levels, particularly those with conditions such as hypogonadotropic hypogonadism, have demonstrated improved outcomes in ART following exogenous LH supplementation [139]. Generally, low levels of endogenous LH have been associated with poorer outcomes in ART [140]. Additional research has shown that fertilization, implantation and clinical pregnancy rates were higher in patients undergoing ART with recombinant human LH (r-hLH) compared to those without r-hLH, along with a lower rate of apoptosis observed in the r-hLH group [141]. It has also been documented that LH supplementation during the mid-follicular phase improves pregnancy outcomes in women who do not respond optimally to conventional ART [137].

Despite the demonstrated benefits of LH supplementation during ART, studies have also indicated that elevated LH levels may adversely affect treatment outcomes [139]. These effects are believably attributed to impaired granulosa cell proliferation, follicular atresia and premature luteinization of preovulatory follicles [139]. Furthermore, increased LH levels prior to ovulation have been shown to negatively impact conception and implantation rates [137].

#### 2.5.2. Male

Similar to females, LH contributes to the maturation of primordial germ cells. In males, LH regulates testosterone synthesis by the Leydig cells of the testes [142]. During puberty, increased LH secretion is essential for the initiation and maintenance of spermatogenesis through Leydig cell maturation and sustained high intratesticular testosterone levels [143].

Measuring LH, along with FSH, is the primary method used to distinguish between primary and secondary hypogonadism [144]. Primary hypogonadism originates from dysfunction within the testes, whereas secondary hypogonadism results from disrupted hormonal signalling between the hypothalamus, pituitary gland and testes [144]. In males with hypogonadotropic hypogonadism, a form of secondary hypogonadism, defective GnRH signalling causes insufficient release of FSH and LH, which leads to decreased androgen levels and reduced spermatogenesis [145]. Studies have shown that treatment with pulsatile GnRH or gonadotropin therapy using LH activity, such as human chorionic gonadotropin (hCG), in combination with FSH can improve spermatogenesis and increase sperm concentration in the ejaculate [146].

The importance of proper LH secretion is further demonstrated in men with end-stage renal disease, who exhibit altered testosterone concentrations associated with elevated plasma LH levels but reduced pulsatile LH secretion compared with healthy individuals or men who have undergone successful renal transplantation. This finding is significant because pulsatile LH release is necessary for normal gonadotropin receptor function in the testes, whereas chronically elevated LH levels may lead to desensitization and loss of gonadotropin receptors, ultimately contributing to testicular dysfunction [147].

### 2.6. Prolactin

#### 2.6.1. Female

Prolactin is a polypeptide hormone mainly produced in the anterior pituitary gland; however, the central nervous system, the immune system, the uterus and the mammary glands are all capable of producing prolactin as well [148]. Prolactin is best known for its role in lactation and breast development; however, emerging research suggests that it also plays a more complex role in reproduction. Studies have demonstrated beneficial effects of prolactin on oocyte development corpus luteum formation and survival, implantation, steroidogenesis and immunomodulation [149–159]. Disruptions in prolactin balance can significantly affect female fertility and the menstrual cycle. In females, elevated prolactin levels inhibit GnRH release, which can disrupt oestrogen and progesterone production, suppress or stop ovulation and lead to irregular or missed periods (amenorrhea) [148,160]. An abnormally high level of prolactin in the blood, known as hyperprolactinemia, is relatively common in women of reproductive age and may cause infertility or galactorrhea, although some individuals remain asymptomatic [148,160,161].

Regardless of the cause of excess prolactin, infertility can occur owing to prolactin-mediated inhibition of GnRH secretion. Dopamine agonists, such as bromocriptine and cabergoline, are commonly used to lower prolactin levels [148,162]. Treatment also involves identifying and addressing the underlying cause, which may include discontinuing a causative medication or treating conditions such as hypothyroidism. Hypothyroidism may present with galactorrhea because elevated thyrotropin-releasing hormone stimulates prolactin secretion [161].

#### 2.6.2. Male

Characteristically, males have low prolactin levels; thus, any elevation suggests an underlying pathological or iatrogenic cause, such as a pituitary adenoma or medication effect [163]. In males, elevated prolactin disrupts the HPG axis, leading to reduced GnRH secretion, decreased spermatogenesis and hypogonadism [164–166]. Clinically, this may present with reduced libido, erectile dysfunction, infertility and occasionally headaches [164]. Mild elevations of prolactin may be asymptomatic, whereas higher levels are more strongly associated with impaired reproductive function [164]. Although hyperprolactinaemia is found in up to 11% of infertile men, it is frequently underdiagnosed owing to its subtle or non-specific clinical manifestations [164,167]. Treatment of elevated prolactin levels in males depends on the underlying aetiology and the presence and severity of symptoms. However, as in females, dopamine agonists such as bromocriptine or cabergoline are commonly used to inhibit prolactin secretion and treat conditions associated with hyperprolactinemia [168].

### 2.7. Androgens

#### 2.7.1. Female

Androgens are steroid hormones essential for human sexual development and reproduction, with broad effects on bone, muscle, adipose tissue, skin, hair, the brain and the cardiovascular system, influencing growth, body composition and behaviour [169]. In females, androgens are produced in the ovaries and adrenal glands and also serve as key precursors for oestrogen synthesis in both ovarian and peripheral tissues, making them essential for normal reproductive function [169]. There are five types of androgens in females, including dehydroepiandrosterone sulphate, dehydroepiandrosterone (DHEA), androstenedione (A_4_), testosterone and dihydrotestosterone (DHT) [170]. Androgens are important for normal follicle development, particularly during the early stages of folliculogenesis, before later serving as substrates for oestrogen production [171]. Additionally, they help regulate communication between follicles and ovarian stromal tissue [171,172]. However, excess androgen levels, as seen in PMOS/PCOS, can disrupt follicular development and ovulation, leading to follicular arrest and infertility [171]. Emerging evidence suggests that androgen supplementation, such as DHEA or testosterone, may improve ovarian response and oocyte yield in some women with low ovarian reserve, potentially enhancing fertility outcomes [171,173–179]. In a randomized, open-label trial of 33 women with low ovarian reserve, daily administration of DHEA was associated with a higher oocyte yield and a significantly increased birth rate [171,176].

#### 2.7.2. Male

In males, androgens are produced primarily in the adrenal glands and the Leydig cells in the testes, with testosterone serving as the principal circulating androgen and playing a crucial role in spermatogenesis and male reproductive function [169]. Androgens are essential for the development and maintenance of male reproductive organs, including the epididymis, vas deferens, seminal vesicle (SV), prostate and the penis, as well as for puberty, male fertility and male sexual function [180]. Furthermore, androgens are needed for muscle growth, bone mineralization, metabolism and cognitive function [180].

Testosterone acts within Sertoli cells by binding to androgen receptors, which are essential for initiating and maintaining spermatogenesis and preventing germ cell apoptosis [21,180]. These receptors are present throughout the male reproductive system and can be activated by testosterone or its more potent metabolite, DHT [180]. Defects in androgen receptor activity or low testosterone levels (hypogonadism) can impair male sexual development and fertility, although spermatogenesis is primarily regulated by gonadotropins and intratesticular testosterone rather than circulating testosterone levels [180,181]. However, low testosterone can reduce sperm production and indirectly impair fertility through decreased libido and erectile dysfunction, which may limit sexual reproduction [181]. Contributing factors to low testosterone include ageing, stress, alcohol and marijuana use, chronic opioid use, obesity, diabetes, testicular injury, kidney disease, cancer treatments and anabolic steroid abuse [180]. Chronic anabolic steroid use, specifically, is a common cause of male infertility, often resulting in oligozoospermia or azoospermia with impaired sperm motility and morphology [180,182]. Although sperm quality often recovers within months after discontinuation, suppression of spermatogenesis may persist for years, alongside low serum testosterone and low FSH and LH levels [180,183]. Importantly, treating low testosterone for males trying to conceive requires avoiding standard testosterone replacement therapy, which suppresses sperm production [180]. Alternatively, males should use medications that boost natural testosterone production, such as clomiphene citrate, hCG or anastrozole [180,182]. Lifestyle changes such as quitting smoking and losing weight may help balance testosterone levels organically [184].

### 2.8. Progesterone

#### 2.8.1. Female

Progesterone is an endogenous steroid hormone that is produced by the adrenal cortex as well as the gonads. In females, progesterone is secreted in the ovaries by the corpus luteum following ovulation. It plays a critical role in female reproductive function by preparing and maintaining the endometrium for embryo implantation and early pregnancy [185,186]. During the luteal phase of the female menstrual cycle, rising progesterone levels promote endometrial receptivity and support implantation of a fertilized embryo. Progesterone is essential for the establishment and maintenance of pregnancy until the placenta becomes competent to secrete sufficient progesterone at approximately 8–10 weeks of pregnancy [185,186]. If the corpus luteum regresses prematurely, before placental function is fully established, spontaneous pregnancy loss occurs [187,188]. If fertilization and implantation do not occur, progesterone concentrations decrease, leading to endometrial shedding and menstruation.

Given progesterone's essential role in endometrial receptivity, embryo implantation and support of early intrauterine pregnancy, impaired luteal progesterone secretion has long been proposed as a potential contributor to infertility and pregnancy loss. This concept underlies luteal phase deficiency (LPD), a condition characterized by inadequate progesterone production or abnormal endometrial response during the luteal phase [189]. Although some studies have reported higher midluteal progesterone and oestrogen concentrations in conception cycles compared with non-conception cycles, findings remain inconsistent and early embryonic hCG signalling may influence these hormonal changes [187,190]. Furthermore, other investigations have demonstrated similar luteal hormone profiles in cycles resulting in successful pregnancy and those ending in early pregnancy loss [187,191,192].

LPD has been associated with infertility, subfertility, recurrent early pregnancy loss, shortened menstrual cycles and premenstrual spotting; however, it has also been identified in ovulatory cycles of otherwise normally menstruating women [22,159, 193–200]. Consequently, the independent role of abnormal luteal function in implantation failure and early pregnancy loss remains controversial.

Pathologic disruption of GnRH and LH pulsatility has been hypothesized to contribute to LPD. Conditions associated with LPD include hypothalamic dysfunction (hypothalamic amenorrhea, eating disorders, excessive exercise, weight loss and stress), metabolic and endocrine disorders (obesity, thyroid dysfunction, hyperprolactinemia and 21-hydroxylase deficiency), reproductive conditions (polycystic ovary syndrome and endometriosis) as well as ageing and exposure to ovarian stimulation or ART [14–16,18–20,187,201–211]. Despite incomplete understanding of causality, the correction of underlying medical conditions may improve luteal oestrogen and progesterone secretion. Women with infertility and suspected abnormal luteal function should be evaluated for underlying medical conditions and treated appropriately based on any identified abnormalities.

Although the role of luteal dysfunction in infertility remains debated, progesterone continues to have important clinical utility as an indirect biomarker of ovulation and luteal function, particularly through assessment of midluteal progesterone levels [23]. Because progesterone secretion reflects corpus luteum activity following ovulation, its measurement can help confirm ovulation and provide insight into endometrial support during the implantation window. However, the significant pulsatility and cycle-to-cycle variability of progesterone secretion, combined with inconsistent associations between luteal progesterone levels and reproductive outcomes, limit its predictive value as a standalone biomarker of fertility or pregnancy success [201].

## 3. Genetic associations with fertility

While endocrine biomarkers provide valuable systemic indicators of HPG axis function in both males and females, they do not fully capture the underlying molecular events within the gonads. To address this limitation, recent studies have utilized transcriptomic analyses and network-based studies to identify key regulatory gene modules that govern gametogenesis at the cellular level and define functional ovarian competence.

### 3.1. Female

In females, these gene networks regulate oocyte development, folliculogenesis and meiotic competence within the ovarian reserve. Within this framework, female reproductive function can be further resolved into discrete gene networks that govern oocyte specification, follicle establishment and maintenance of the ovarian reserve.

A central group of genes acts as upstream regulators of germ cell identity and early follicular development, coordinating transcriptional programs required for germ cell differentiation and primordial follicle formation. Key examples include *FIGLA*, *LHX8*, *NOBOX*, *SOHLH1* and *SOHLH2*, which orchestrate oocyte-specific gene expression programs required for primordial follicle formation and early oocyte differentiation [24–30]. Additional factors such as *TAF4B* contribute to germ cell survival and transcriptional regulation within the developing oocyte [24,31]. In contrast, genes such as *FOXO3* function primarily as regulators of primordial follicle dormancy and activation, maintaining the quiescent state of the ovarian reserve [32].

A second group of genes are essential for ovarian differentiation, granulosa cell identity and the structural and endocrine framework required for folliculogenesis. These genes include *FOXL2*, *WT1*, *WNT4* and *NR5A1* [33–37]. While not oocyte-intrinsic, these factors are critical for creating and maintaining the cellular environment that supports oocyte maturation.

Downstream of these lineage-defining regulators, additional signalling pathways mediate bidirectional communication between the oocyte and surrounding somatic cells and integrate endocrine cues. Key components include *AMH* and *FSHR*, which regulate follicle recruitment and gonadotropin responsiveness, and *CYP19A1*, which drives oestrogen biosynthesis in granulosa cells [38–42]. Oocyte-derived factors such as *GDF9* and *BMP15* are essential for granulosa cell proliferation and follicular progression [212–214]. Additional mediators, including *KIT,* support oocyte survival and growth [215]. Collectively, these pathways integrate intra-ovarian signalling with systemic hormonal regulation to coordinate follicle activation, oocyte competence and reproductive lifespan.

While these regulatory and signalling networks establish oocyte and follicular development, successful female fertility ultimately depends on the integrity of meiotic processes that ensure accurate chromosome segregation, DNA repair and oocyte genomic stability. As such, variants in meiosis-associated genes are increasingly recognized as important contributors to female infertility. Many of these variants are exceedingly rare or absent from population databases, likely because they cause severe infertility phenotypes that markedly reduce reproductive fitness, such as oocyte maturation arrest caused by *CDC20* variants [216,217]. In contrast, more prevalent variants are often associated with less severe, yet still clinically significant, reproductive outcomes, including increased susceptibility to premature ovarian insufficiency (POI), DOR, elevated rates of oocyte aneuploidy and accelerated ovarian ageing [35,216,218–246]. Collectively, studies of meiosis-associated genes involved in meiotic recombination and DNA repair (including *BRCA1, BRCA2, DMC1, ERCC6, HFM1, MCM8, MCM9, MEIOB, MLH3, MSH4, MSH5, NBN, PSMC3IP, SPO11* and *XRCC4*), chromosome synapsis and cohesion (including *REC8, SMC1B, STAG3, SYCE1, SYCP2* and *SYCP3*), chromosome segregation and spindle regulation (including *AURKB, AURKC, BUB1B, CEP120, CKAP5, SGO2, TRIP13, TUBB8* and *WEE2*) and oocyte maturation and germ-cell maintenance (including *EIF4ENIF1, NANOS3, NUP107, PATL2, POF1B, STRA8* and *YBX2*) underscore the critical importance of meiotic integrity for the generation of euploid, developmentally competent oocytes and female fertility [35,216,218–221,223–237,239–248].

### 3.2. Male

In males, these gene networks regulate spermatogonial maintenance, meiotic progression, spermiogenesis and the production of functionally competent spermatozoa within the seminiferous epithelium. Within this framework, male reproductive function can similarly be resolved into discrete gene networks that govern germ-cell specification, spermatogonial differentiation, meiotic integrity and post-meiotic sperm maturation.

A central group of genes function in the maintenance and differentiation of spermatogonial stem cells and early germ-cell development, coordinating transcriptional programs required for spermatogenesis initiation and progression. Key regulators include *DAZ, DAZL, DDX4, DMRT1* and *NANOS2*, which are essential for germ-cell survival, spermatogonial maintenance and differentiation [249–252]. Additional transcriptional regulators such as *NR5A1* contribute to testicular development, Sertoli cell function and endocrine regulation of spermatogenesis, while *SOX9* and *WT1* are critical for testis differentiation and maintenance of the seminiferous microenvironment required to support germ-cell development [253,254]. In contrast, genes including *KIT* and *KITLG* primarily regulate spermatogonial proliferation, survival and communication between germ cells and their surrounding testicular microenvironment [255,256].

A second group of genes is essential for Sertoli-germ cell communication, hormonal responsiveness and structural support of spermatogenesis. These include *AR, FSHR* and *LHCGR*, which mediate androgen and gonadotropin signalling pathways necessary for testicular maturation and sperm production [257,258]. Additional regulators, including cystic fibrosis transmembrane conductance regulator (*CFTR),* contribute to male reproductive tract development and sperm transport, with pathogenic variants commonly associated with congenital bilateral absence of the vas deferens and obstructive azoospermia [259]. Genes such as *TEX14* and *SUN5* additionally contribute to intercellular bridge formation and sperm structural integrity during spermiogenesis, underscoring the importance of coordinated cellular architecture in sperm maturation [260,261]. Collectively, these pathways integrate endocrine signalling with local testicular regulatory networks to support continuous sperm production and maturation throughout reproductive life.

While these regulatory and signalling networks establish spermatogonial development and spermatogenesis, successful male fertility ultimately depends on the integrity of meiotic processes that ensure accurate chromosome synapsis, homologous recombination, DNA repair and chromosome segregation. Consequently, variants in meiosis-associated genes are increasingly recognized as important contributors to male infertility, particularly non-obstructive azoospermia and spermatogenic arrest [262]. Similar to female infertility-associated variants, many pathogenic variants affecting meiotic genes are exceedingly rare or absent from population databases owing to their severe effects on reproductive fitness. In contrast, more prevalent variants may contribute to milder but clinically significant phenotypes including oligozoospermia, teratozoospermia, asthenozoospermia, elevated sperm DNA fragmentation and increased sperm aneuploidy [263].

Collectively, studies of meiosis-associated genes involved in synaptonemal complex formation and chromosome synapsis (including *SYCP2, SYCP3* and *SYCE1*), meiotic recombination and DNA repair (including *DMC1, MSH4, MSH5, MLH1, SPO11* and *RAD21L1*), meiotic checkpoint regulation and chromosome cohesion (including *HORMAD1, STAG3, TEX11* and *REC8*) and post-meiotic spermatid differentiation and sperm structural integrity (including *DPY19L2, SUN5, SEPT12* and *AURKC*) underscore the critical importance of meiotic fidelity and coordinated germ-cell development for the generation of genetically stable, functionally competent spermatozoa and maintenance of male fertility [262,264–272].

## 4. Genetic and epigenetic markers of infertility

Genetic and epigenetic biomarkers have emerged as increasingly important components of fertility assessment, providing insight into the molecular mechanisms underlying reproductive competence, gametogenesis, embryonic development and infertility-related disorders [273]. While traditional clinical evaluations primarily assess anatomical, endocrine and physiological function, genetic analyses enable the identification of inherited variants, chromosomal abnormalities and mutations that may impair reproductive potential in both males and females [273]. Alongside this, recent advances in multi-omics, including genomics, transcriptomics, epigenomics, proteomics, metabolomics and microbiomics, are increasingly being integrated into reproductive medicine, expanding the diagnostic framework beyond conventional clinical assessment [274,275].

### 4.1. Female (genetics)

In females, genetic abnormalities contribute substantially to reproductive dysfunction, including conditions such as PMOS/PCOS, DOR, disrupted folliculogenesis, recurrent pregnancy loss and POI [273,274]. Chromosomal aneuploidies and structural abnormalities involving the X chromosome are major contributors to female infertility [35,273]. Conditions such as Turner syndrome and X chromosome deletions frequently result in accelerated oocyte depletion, ovarian dysfunction and gonadal dysgenesis [273]. In Turner syndrome patients, altered expression and methylation patterns have been reported in genes such as *BMP15*, *KDM6A*, *ZFX* and *USP9X* [273,276,277].

Beyond chromosomal alterations, single-gene defects affecting oocyte development and folliculogenesis play a central role in ovarian dysfunction. Variants in genes including *FMR1, BMP15, GDF9, NOBOX, FIGLA* and *PGRMC1* have been associated with impaired follicular development, DOR and POI [35]. Notably, CGG repeat expansions in the 5′ untranslated region of *FMR1* are strongly associated with DOR and fragile X-associated POI, reflecting its functional relevance in granulosa and germ cell populations [35].

Defects in pathways governing meiosis, DNA repair and genomic stability further contribute to reproductive impairment. Variants in genes such as *BRCA1, BRCA2, MSH4, MSH5* and *STAG3* may compromise homologous recombination and oocyte integrity, accelerating reproductive ageing through the accumulation of DNA damage [35]. In addition, mitochondrial DNA mutations, such as in the *POLG* gene, and mitochondrial dysfunction have been implicated in reduced oocyte competence, reflecting the high energetic demands of oogenesis and early embryonic development [35].

Extending beyond single-gene and pathway-level analyses, network-based bioinformatics approaches have been widely applied to investigate the molecular architecture of female infertility. These studies have identified putative hub genes within disease-associated interaction networks, although results often vary depending on dataset, methodology and analytical framework. For example, *UPF3B*, *IRF8* and *PSMB1* were reported as central nodes in one infertility-associated network analysis [278]. Overall, these findings suggest that female infertility is influenced by interconnected gene networks rather than single genes acting alone, and that key “hub” genes vary depending on the study and biological context.

### 4.2. Male (genetics)

In males, genetic factors play well-recognized roles in infertility, with Y chromosome abnormalities being especially important [279–281]. Major genetic contributors to male infertility include chromosomal abnormalities and Y chromosome microdeletions, both of which are strongly associated with azoospermia and severe oligozoospermia [282]. Among chromosomal disorders, karyotypic abnormalities, particularly Klinefelter syndrome, represent the most common sex chromosome abnormalities linked to male infertility [283,284]. In addition, Kallmann syndrome, a genetic disorder characterized by hypogonadotropic hypogonadism and impaired GnRH signalling, can lead to delayed puberty and severely reduced sperm production as a result of insufficient hormonal stimulation of the testes [285]. Microdeletions within the azoospermia factor regions of the Y chromosome are clinically significant and constitute the most common structural chromosomal abnormalities associated with impaired spermatogenesis and male infertility [279].

Certain inherited disorders are also associated with male infertility. For example, mutations in the *CFTR* gene are strongly linked to *CBAVD*, a condition that causes obstructive azoospermia despite otherwise preserved spermatogenesis [285].

Consequently, genetic screening for *CFTR* variants is routinely recommended in affected individuals genetic consulting prior to assisted reproduction owing to the potential risk of transmission to offspring [279,285].

As mentioned in the previous section, additional infertility-associated genes include *TEX11*, *SYCP3*, *NR5A1*, *DMRT1* and *DAZ*, many of which regulate germ cell differentiation, meiotic recombination or Sertoli cell function [36,249,252,262].

### 4.3. Female (epigenetics)

Beyond inherited genetic variants, epigenetic regulation has emerged as a critical determinant of reproductive function. Epigenetic modifications including DNA methylation, histone modification, chromatin remodelling and non-coding RNA (ncRNA) regulation govern gene expression patterns essential for gametogenesis, embryonic development, genomic imprinting and reproductive ageing [286–288]. Epigenetics involves stable or heritable changes in gene expression without alteration of the DNA sequence, and disruption of these processes may impair gamete quality despite the absence of overt genetic mutations.

In females, aberrant DNA methylation patterns within oocytes and granulosa cells have been associated with DOR, POI, PMOS/PCOS and implantation failure [288,289]. Beyond DNA methylation abnormalities, RNA methylation also plays important roles in ovarian function and follicular fate by regulating RNA stability, transport, splicing and translation at the post-transcriptional level. Altered RNA methylation has been linked to reproductive disorders including preeclampsia, miscarriage, endometriosis and POI [286]. Age-related epigenetic changes additionally contribute to declining oocyte quality and increased embryonic aneuploidy observed with reproductive ageing [289,290].

### 4.4. Male (epigenetics)

In males, sperm epigenetic integrity is increasingly recognized as an important determinant of reproductive potential. Significant associations between DNA methylation and sperm quality have been linked to sperm motility, morphology and DNA integrity [291–293]. Notably, aberrant DNA methylation of imprinted genes has been proposed as an important contributor to impaired spermatogenesis and male infertility [291,294].

Histone modifications also play essential roles throughout spermatogenesis, fertilization and early embryonic development [291,295]. Numerous studies have demonstrated that abnormal histone remodelling during spermatogenesis can negatively affect sperm production and male fertility [291,296]. In addition, emerging evidence has expanded the definition of epigenetic regulation to include mechanisms mediated by ncRNAs, which are capable of modulating gene expression without altering the underlying DNA sequence [291,297].

Environmental factors, such as endocrine-disrupting chemicals and lifestyle elements, have the potential to affect germline epigenetic markers, with sperm cells being the ultimate recipients of these changes [201].

Environmental and lifestyle factors, including exposure to endocrine-disrupting chemicals, smoking, diet and stress, may further influence germline epigenetic profiles, with spermatozoa serving as the final carriers of these modifications [291,298]. Abnormal DNA methylation patterns, altered histone retention and dysregulated sperm-borne small ncRNAs have all been associated with reduced sperm motility, abnormal morphology, impaired fertilization, recurrent miscarriage and adverse embryonic outcomes [299]. Because mature sperm contribute not only paternal genetic material but also epigenetic information required for early embryonic regulation, epigenetic abnormalities may influence both male fertility and offspring development.

Emerging high-throughput technologies, including next-generation sequencing, genome-wide association studies, single-cell transcriptomics and methylation profiling, continue to expand understanding of infertility-associated molecular pathways [275]. These approaches are increasingly being integrated into precision reproductive medicine to improve diagnostic accuracy, identify novel therapeutic targets and optimize patient-specific fertility treatment strategies.

Together, genetic and epigenetic biomarkers provide critical insight into the molecular architecture of reproductive health. By complementing endocrine, anatomical and clinical assessments, these markers enhance the ability to identify underlying causes of infertility, predict reproductive outcomes and guide individualized approaches to fertility management and assisted reproduction.

## 5. Gamete-based functional assessment

Gamete-based functional assessment examines the physiological and developmental competence of mature gametes as operative units of reproduction. In contrast to genetic, epigenetic or systemic approaches that interrogate upstream regulatory or etiological mechanisms, this section emphasizes quantifiable outcomes that determine fertilization potential and early embryonic development.

### 5.1. Female

Female gamete functional assessment evaluates oocyte developmental competence, or the ability of an oocyte to support fertilization and sustain healthy embryonic development. Rather than focusing on upstream regulatory pathways, this approach emphasizes measurable functional characteristics and downstream reproductive outcomes. Oocyte quality is routinely assessed through morphological evaluation and classification of the oocyte and its associated structures, including cumulus cells, the zona pellucida, polar body, perivitelline space and cytoplasm, typically using conventional microscopy [300]. Progression to the metaphase II stage is required for fertilization competence; however, morphological criteria alone provide only limited predictive value for subsequent developmental success [300].

Beyond morphology, oocyte competence is also supported by a range of functional characteristics, including mitochondrial energy production, cytoplasmic maturation and calcium signalling dynamics [300,301]. The functional integrity of the oocyte is ultimately reflected in downstream reproductive outcomes, particularly fertilization success and embryonic viability. Disruptions in chromosomal segregation during oocyte development can result in aneuploid embryos, which are strongly associated with implantation failure and miscarriage [302]. Insufficient oocyte competence may also manifest as failed fertilization or early embryonic arrest, reflecting inadequate developmental support for embryo progression [303].

In addition to morphological and functional assessment, emerging biomarkers are being investigated for their ability to more precisely predict oocyte competence. These include indicators of mitochondrial performance, cumulus cell gene expression profiles and follicular fluid metabolomic signatures [300]. Together, these biomarkers provide a more integrated reflection of oocyte functional status and are increasingly explored for their predictive value in reproductive outcomes.

### 5.2. Male

Semen analysis remains the primary clinical tool for assessing male reproductive potential. It provides a basic characterization of ejaculate quality; however, it has limited sensitivity for detecting functional defects affecting fertilization and early embryonic development [304,305].

The conventional parameters of sperm concentration, motility and morphology form the basis of this assessment. Sperm concentration reflects spermatogenic output, motility indicates progressive movement capability and morphology evaluates structural integrity [306]. While collectively informative, these measures do not fully capture fertilization competence.

To address this limitation, functional sperm assays evaluate biological competence beyond conventional semen parameters. These include membrane integrity, chromatin integrity, capacitation capacity, acrosome reaction potential, mitochondrial activity and sperm–oocyte interaction ability [306–308]. Together, these parameters provide a more direct measure of fertilization potential.

At the molecular level, sperm DNA integrity represents the cumulative impact of cellular stress during sperm development and maturation and is a key determinant of reproductive outcomes [309]. The DNA fragmentation index (DFI) quantifies the proportion of spermatozoa exhibiting DNA strand breaks. Elevated DFI has been associated with reduced fertilization rates, impaired embryonic development and an increased risk of pregnancy loss. However, the extent to which DFI independently predicts clinical outcomes, particularly miscarriage risk, remains controversial, with inconsistent findings across studies [310–312].

Complementing this, abnormal chromatin packaging reflects incomplete nuclear remodelling during spermiogenesis, characterized by inadequate histone-to-protamine substitutions and reduced chromatin condensation [313]. These defects compromise genomic stability, increasing susceptibility to oxidative and endogenous damage and have been clinically associated with reduced fertilization rates and abnormal embryonic development [313–316].

Beyond DNA- and chromatin-based measures, emerging biomarkers provide molecular-level indicators of sperm functional competence beyond standard semen analysis. These include sperm RNA content, proteomic signatures, mitochondrial function markers and metabolomic profiles of seminal plasma and extracellular vesicles [317,318]. These markers reflect downstream functional states of spermatozoa and are being investigated for their association with fertilization outcomes and embryo development.

## 6. Imaging assessments

Imaging modalities play a central role in the investigation of infertility by providing structural and functional assessment of the reproductive organs in both males and females. Among these modalities, ultrasonography remains the primary first-line imaging technique owing to its non-invasive nature, accessibility, low cost, lack of radiation and ability to provide real-time visualization of reproductive anatomy. In its basic form, ultrasound is an imaging technique that generates images by transmitting high-frequency sound waves into the body and detecting their echoes as they reflect off tissues with differing acoustic impedance, which are then processed by a receiver to produce a two-dimensional image [319]. Ultrasound-based assessments are routinely integrated with endocrine, genetic and clinical evaluations to identify anatomical abnormalities, monitor gametogenesis and guide assisted reproductive interventions.

### 6.1. Female

In females, transvaginal ultrasonography (TVUS) is considered the standard imaging approach for evaluating pelvic reproductive anatomy and ovarian function [320]. TVUS enables high-resolution visualization of the uterus, ovaries, endometrium and adnexal structures, allowing clinicians to identify abnormalities associated with infertility such as uterine fibroids, congenital Müllerian anomalies, endometrial polyps, adenomyosis, uterine synechiae, hydrosalpinx and pyosalpinx and ovarian cysts [321–325]. Ultrasonography is also essential for assessing ovarian reserve through (AFC), which estimates the number of available follicles within the ovaries and serves as an important predictor of reproductive potential and responsiveness to ovarian stimulation [326]. In addition to ovarian reserve assessment, TVUS plays a central role in the evaluation of disorders that affect follicular development and ovarian morphology, particularly PMOS/PCOS, according to current diagnostic criteria, and ovarian hyperstimulation syndrome (OHSS), both of which are commonly encountered in reproductive medicine [327,328]. Additionally, ultrasonographic monitoring is widely used during ART to assess follicular growth, evaluate endometrial development and receptivity for implantation and guide oocyte retrieval and embryo transfer procedures, and monitor patients for excessive ovarian response and the development of OHSS [327,329].

Specialized ultrasound techniques further enhance reproductive evaluations. For instance, Doppler ultrasonography evaluates vascular perfusion within the uterus and ovaries, providing insight into endometrial receptivity and ovarian blood flow [330]. Saline infusion sonohysterography (SIS), also referred to as sonohysterography or hysterosonography, is widely utilized as a minimally invasive screening tool for evaluating the uterine cavity [331]. During the procedure, sterile saline is infused transcervically through a catheter into the uterine cavity, allowing enhanced visualization of the endometrial lining and overall uterine cavity contour. This enables the detection of structural abnormalities, such as polyps, septa, fibroids, adhesions or congenital uterine anomalies that may impair implantation or pregnancy outcomes [331]. Hysterosalpingo-contrast sonography (HyCoSy) is commonly performed concurrently with SIS to additionally evaluate fallopian tube patency through the administration of echo-enhancing agents [332]. As a first-line diagnostic approach for tubal assessment, HyCoSy is considerably less invasive than laparoscopic and salpingoscopic procedures while still providing valuable information regarding tubal patency and reproductive tract anatomy [333].

Additional imaging modalities may be utilized when ultrasonographic findings are inconclusive or when more detailed anatomical characterization is required. Among these modalities, hysterosalpingography is an alternative radiographic technique used to evaluate the uterine cavity and fallopian tube patency; however, the procedure may cause patient discomfort and requires the use of contrast and radiation [334]. Magnetic resonance imaging (MRI) may also be used, which provides superior soft tissue resolution and is especially valuable in the evaluation of complex Müllerian anomalies, deep infiltrating endometriosis, adenomyosis and pelvic masses [335–338]. In select cases, computed tomography may be employed for broader pelvic or abdominal evaluation, although its routine use in infertility assessment is limited owing to radiation exposure and reduced soft tissue contrast compared to MRI [320].

### 6.2. Male

In males, scrotal ultrasonography is the primary imaging modality for evaluating testicular structure and associated reproductive pathologies [339]. Imaging may also assist in surgical planning and sperm retrieval procedures in patients undergoing fertility preservation or assisted reproduction. High-frequency ultrasound enables detailed assessment of testicular size, echotexture and vascular perfusion while also identifying abnormalities such as varicocele, hydrocele, epididymal cysts, intratesticular and extratesticular masses and other testicular or scrotal abnormalities involving the epididymis and vas deferens [339]. Doppler ultrasonography, another evaluation tool, has a sensitivity of almost 100% in detecting acute inflammation [339]. It is thus a well-established imaging technique for the diagnosis of epididymitis and is particularly important in the diagnosis of varicoceles, which represent one of the most common correctable causes of male infertility [340]. Imaging findings may also reveal hydrocele or pyelocele, scrotal wall thickening, fistula formation and calcifications [339,341–344].

Vasography is a diagnostic imaging modality historically used to investigate disorders of the male genital tract, including causes of azoospermia, persistent perineal pain, seminal vesiculitis and prostate cancer [345]. Traditionally, the procedure involves puncture or partial-thickness vasotomy of the scrotal vas deferens, followed by retrograde contrast injection to evaluate ductal patency, with passage of contrast into the bladder indicating an unobstructed pathway [346]. Although long considered the gold standard for evaluating the vas deferens and ejaculatory ducts, vasography is an invasive technique that often requires anaesthesia and carries risks including infection, vasal stricture formation and injury at the puncture site [17,346,347]. Consequently, its use has declined substantially with the widespread adoption of less invasive imaging modalities such as ultrasonography, MRI and transrectal ultrasonography (TRUS) [346,347].

Similarly, venography has traditionally been regarded as the gold standard for diagnosing varicocele through visualization of retrograde contrast reflux into the testicular vein [346,348]. Owing to its invasive nature, venography is now generally reserved for patients undergoing therapeutic embolization of the testicular vein after varicocele diagnosis has been established clinically and with ultrasound [346].

More recently, TRUS has emerged as an important minimally invasive imaging method for the evaluation of ejaculatory duct obstruction and azoospermia, particularly in patients presenting with low ejaculate volume [347,349–354]. TRUS allows visualization of the prostate, SV and ejaculatory ducts, facilitating identification of obstructive abnormalities, congenital absence of the vas deferens, congenital SV defects, SV dilation and cysts [349].

Collectively, ultrasound and imaging assessments provide critical structural and functional information that complements molecular, endocrine and clinical biomarkers of reproductive health. By enabling the visualization of anatomical structures, evaluation of reproductive organ function and monitoring of therapeutic interventions, imaging modalities remain indispensable components of modern infertility diagnosis and management in both males and females.

## 7. Inflammatory and metabolic determinants of reproductive function

Reproductive function is increasingly understood as being tightly integrated with systemic metabolic and inflammatory status. Rather than operating in isolation, the HPG axis is highly sensitive to circulating cytokines, adipokines, metabolic hormones and oxidative stress mediators [355,356]. Disruptions in these systems can impair gametogenesis, steroidogenesis, endometrial receptivity and early embryonic development, contributing to a broad spectrum of infertility phenotypes in both males and females [355,356].

A central feature of this dysregulation is chronic low-grade inflammation, which is frequently observed across infertility-associated conditions. Elevated systemic inflammatory markers, including C-reactive protein, interleukin-6, tumour necrosis factor-alpha and related cytokines, have been associated with impaired reproductive outcomes [357,358]. These mediators can disrupt ovarian follicular development, alter endometrial receptivity and negatively affect spermatogenesis through immune–endocrine crosstalk [357,358]. In females, chronic inflammation can interfere with oocyte quality, folliculogenesis, hormone production and immune signalling while also impairing implantation through altered endometrial immune tolerance [359]. In males, systemic inflammation has been associated with reduced sperm concentration, impaired sperm motility and morphology and increased sperm DNA damage, reflecting disruption of the testicular microenvironment and germ cell integrity [358].

Closely linked to inflammatory dysregulation is oxidative stress, defined as an imbalance between reactive oxygen species (ROS) production and antioxidant defence systems [358,360,361]. While physiological ROS levels are required for processes such as ovulation, endometrial remodelling, capacitation and sperm function, excessive oxidative stress is strongly associated with reproductive dysfunction [358,362,363,364]. In oocytes, oxidative stress can induce mitochondrial dysfunction, spindle instability, chromosomal abnormalities, telomere shortening and reduced developmental competence of aged oocytes [365]. Further consequences include impaired oocyte competence, impaired endometrial receptivity, higher rates of implantation failure and pregnancy loss and premature exhaustion of the primordial follicle reserve [364,366,367]. In spermatozoa, oxidative damage is particularly detrimental owing to limited antioxidant capacity, leading to lipid peroxidation oxidation, mitochondrial dysfunction and sperm DNA fragmentation, thereby compromising motility, morphology and fertilizing capacity [358]. Oxidative stress also contributes to endometrial dysfunction and early embryonic loss, underscoring its systemic impact across reproductive tissues.

These inflammatory and oxidative pathways are closely intertwined with metabolic dysfunction, particularly in the context of metabolic syndrome. Characterized by impaired glucose metabolism, abdominal obesity, dyslipidaemia and hypertension, metabolic syndrome exerts significant adverse effects on reproductive health in both males and females [368]. Central to its reproductive effects is insulin resistance, which disrupts HPG axis signalling and alters gonadotropin secretion and sex steroid homeostasis [368]. Adipose tissue additionally functions as an important endocrine organ that regulates energy homeostasis and metabolism through the secretion of adipokines [369]. Adipocyte-derived factors, including leptin, adiponectin and resistin, as well as gut-derived peptides, such as ghrelin, play critical roles in linking metabolic status with reproductive function [369]. These factors modulate reproductive signalling at both central and gonadal levels [369]. Within this metabolic–reproductive interface, sex hormone-binding globulin (SHBG) also serves an important regulatory role. SHBG is synthesized predominantly in the liver, and its production is suppressed by hyperinsulinemia and inflammatory signalling [370]. Reduced SHBG concentrations increase the bioavailability of sex steroids, thereby amplifying androgenic and oestrogenic effects on reproductive tissues and contributing to dysregulated feedback within the HPG axis [371].

In males, metabolic syndrome has been linked to reduced circulating testosterone concentrations, suppression of SHBG, enhanced aromatase-mediated conversion of androgens to oestrogens within adipose tissue and impaired spermatogenic function, collectively contributing to a state of functional hypogonadotropic hypogonadism and compromised reproductive capacity [368,372,373].

PMOS/PCOS represents a prototypical reproductive–metabolic disorder in which hyperandrogenism, insulin resistance, chronic inflammation and oxidative stress converge to impair ovarian function and fertility. Hyperinsulinemia plays a pivotal role in PMOS/PCOS pathophysiology by stimulating ovarian theca cell androgen production while simultaneously suppressing hepatic SHBG synthesis [45,374]. Reduced SHBG further increases free androgen bioavailability, intensifying hyperandrogenic features and contributing to follicular arrest [374]. This endocrine environment disrupts normal follicular maturation, leading to anovulation and reduced oocyte quality [374]. Additionally, inflammatory and oxidative stress pathways are frequently upregulated in PMOS/PCOS, contributing to a hostile uterine microenvironment that compromises endometrial receptivity and impairs embryo implantation [375].

Underlying these condition-specific indicators is a broader network of metabolic–reproductive signalling pathways that coordinate energy availability with gonadal function [376]. Key regulatory axes include insulin/insulin-like growth factor-1 signalling, the PI3K/PTEN/Akt pathway, AMP-activated protein kinase (AMPK) and mammalian target of rapamycin, all of which play essential roles in sexual development and reproductive function of both males and females [377–382]. These intracellular signalling pathways are further governed by systemic endocrine signal cues that reflect whole-body energy availability. Leptin acts as an upstream adipocyte-derived signal of systemic energy expenditure, modulating hypothalamic control of the HPG axis and interfacing with intracellular energy-detecting pathways including PI3K/Akt and AMPK [383].

Together, dysregulation of these hormonal and intracellular networks under metabolic stress conditions impairs reproductive capacity, reinforcing the concept that fertility is tightly gated by systemic metabolic health.

## 8. Future directions in fertility assessments

Recent advances in molecular biology and precision medicine have led to increasing interest in the identification of novel biomarkers for infertility diagnosis, prognosis and therapeutic guidance. While conventional fertility assessments primarily rely on hormonal evaluation, imaging and semen analysis, emerging molecular approaches aim to provide a more detailed understanding of reproductive function at the cellular and biochemical levels. In particular, telomere-associated biomarkers, ncRNAs, circulating protein markers and proteomic profiling have shown promise as future tools for improving infertility assessment and ART.

In recent years, a growing body of research has examined the relationship between telomere length and fertility, identifying sperm telomere length as a potential biomarker of male fertility [384]. Telomeres are repetitive nucleotide sequences located at chromosome ends that preserve genomic stability during cell division [385]. Shortened sperm telomere length has been associated with impaired spermatogenesis, oxidative stress, reduced sperm quality and poorer reproductive outcomes [384,385]. In addition to telomere length itself, telomeric repeat-containing RNAs (TERRA) have also been investigated as potential biomarkers of spermatozoal quality owing to their regulatory role in telomere maintenance and chromosomal integrity and TERRA expression was found to be a predictive factor for embryo quality in the setting of assisted reproduction [384].

Non-coding RNAs have emerged as key regulators of reproductive physiology, with Piwi-interacting RNAs (piRNAs) representing a particularly important class in male germ cells [386]. Highly enriched in the testes, piRNAs support spermatogenesis by suppressing transposable elements and maintaining genomic stability [387]. Disruption of piRNA expression has been associated with impaired spermatogenesis and male infertility, and distinct seminal plasma piRNA signatures have been identified in patients with azoospermia and asthenozoospermia [387]. More recently, altered piRNA profiles in seminal plasma have been linked to reduced sperm count and impaired motility, supporting their potential utility in non-invasive infertility assessment [386]. Given their germ cell-specific origin, decreased piRNA levels may also reflect diminished germ cell populations, particularly in azoospermic and oligozoospermic males [386,388].

Similarly, microRNAs (miRNAs) are involved in post-transcriptional gene regulation and play critical roles in ovarian function, folliculogenesis, implantation and early embryonic development [389]. Dysregulated miRNA expression has been linked to conditions such as PMOS/PCOS, endometriosis, DOR and unexplained infertility, highlighting their potential utility as both diagnostic biomarkers and therapeutic targets [389].

Protein-based biomarkers are also being explored in reproductive medicine. For instance, vascular endothelial growth factor (VEGF), a key regulator of angiogenesis and endometrial vascularization, has been investigated as a potential biomarker in female unexplained infertility [390]. Altered serum VEGF levels may reflect abnormalities in endometrial receptivity and implantation potential, although further studies are required to validate its clinical utility [390]. More broadly, advances in proteomic technologies have enabled comprehensive analysis of proteins present in semen, follicular fluid, endometrial secretions and embryo culture media [391–393]. Proteomic profiling may improve assessment of gamete quality, embryo viability and implantation potential while facilitating more individualized ART protocols.

In addition to molecular biomarker discovery, future advances in infertility assessment are increasingly focused on non-invasive diagnostic approaches and continuous reproductive monitoring technologies. Emerging liquid biopsy-based techniques utilizing saliva, urine, menstrual fluid, semen and blood samples may allow for earlier detection of reproductive dysfunction while reducing the need for invasive procedures [394]. Furthermore, wearable technologies and digital health platforms capable of tracking basal body temperature, heart rate variability, sleep patterns, hormonal fluctuations and menstrual cycle characteristics in real time are becoming increasingly integrated into fertility monitoring and reproductive health management [395]. These technologies may improve cycle prediction, optimize timing for conception or assisted reproductive interventions and provide individualized longitudinal reproductive data [395,396]. Although many of these approaches remain under clinical investigation, they reflect a broader transition towards personalized, preventative and data-driven reproductive medicine.

Collectively, these emerging molecular and multi-omics approaches represent a shift towards precision reproductive medicine. However, individual biomarkers may have limited diagnostic or prognostic value when evaluated in isolation. Future fertility assessments will likely require the integration of combinatorial biomarkers that incorporate complementary molecular, clinical and physiological measures to improve predictive accuracy and provide a more comprehensive assessment of reproductive potential. For example, the combination of endocrine markers with genomic, transcriptomic, proteomic or metabolomic signatures may enhance the clinical utility of biomarkers that demonstrate limited value as standalone measures. The integration of genomics, epigenomics, transcriptomics, proteomics and metabolomics may ultimately allow for earlier infertility detection, improved prognostic accuracy and more personalized therapeutic interventions. Furthermore, the increasing availability of large-scale multi-omics datasets creates new opportunities for biomarker discovery through advanced bioinformatic approaches. Beyond traditional analytical pipelines, strategies such as network analyses, unbiased clustering, machine learning approaches and digital twin modelling may enable the identification of novel biomarkers and molecular signatures associated with infertility phenotypes and treatment outcomes [397–400]. Integrating multi-omics datasets with clinical datasets may further improve diagnostic and prognostic capabilities while supporting more individualized fertility assessment and treatment strategies. However, despite promising preliminary findings, many of these biomarkers remain under investigation owing to challenges including limited standardization, small study populations, high analytical complexity and insufficient large-scale clinical validation. Further research will be necessary before these approaches can be routinely incorporated into clinical infertility evaluation and management.

## 9. Conclusions

Overall, emerging evidence supports reproductive function as the product of highly interconnected biological networks that collectively govern gamete quality, developmental competence and fertility outcomes. Rather than operating independently, endocrine, genetic, epigenetic, metabolic, inflammatory and functional pathways converge to shape a dynamic reproductive system that is continuously influenced by both intrinsic and extrinsic factors.

Despite substantial progress in the identification of potential biomarkers and assessment strategies, translation into routine clinical practice remains limited by challenges related to standardization, clinical validation, accessibility, ethical implementation and the lack of large, well-characterized and heterogeneous study populations. Furthermore, many current approaches continue to evaluate isolated parameters, limiting a more integrated understanding of reproductive health and infertility.

Continued integration of molecular, functional and clinical perspectives will be essential for improving diagnostic accuracy, expanding understanding of infertility pathophysiology and optimizing reproductive outcomes. Future progress will likely depend on the integration of multi-omics approaches with robust clinical datasets to facilitate the development of reliable, predictive and clinically actionable biomarkers. Ultimately, these advances may support more comprehensive diagnostic frameworks and increasingly individualized approaches to fertility assessment and treatment.

## Data Availability

This article has no additional data.
